# Investigation of the sensitivity of human A549 cells to paclitaxel and sesquiterpene lactone alantolactone via apoptosis induction

**DOI:** 10.1007/s00210-025-03947-w

**Published:** 2025-02-28

**Authors:** Irem Bayar, Yalcin Erzurumlu, Senem Akkoc, Zafer Bulut, Mehmet Nizamlioglu

**Affiliations:** 1 Department of Biochemistry, Faculty of Veterinary Medicine, Selcuk University, Konya, Turkey; 2 Department of Pharmaceutical Research and Development, Institute of Health Sciences, Suleyman Demirel University, Isparta, Turkey; 3 Department of Basic Pharmaceutical Sciences, Faculty of Pharmacy, Suleyman Demirel University, Isparta, Turkey; 4 Faculty of Engineering and Natural Sciences, Bahcesehir University, Istanbul, Turkey; 5 Department of Biochemistry, Faculty of Veterinary Medicine, Dokuz Eylul University, Izmir, Turkey

**Keywords:** A549, Alantolactone, Apoptosis, Lung cancer, Paclitaxel

## Abstract

Alantolactone (ALA), a sesquiterpene lactone compound obtained from *Inula helenium* root, is known to have anticancer activity in many types of cancer. Paclitaxel (PAX) is an effective first-line chemotherapeutic drug and is widely used in the treatment of lung cancer. The in vitro anticancer efficacy of combined treatment of ALA with PAX was investigated in the A549 human lung cancer cell line. The results show that ALA potentiated the effect of PAX-induced growth restriction and apoptosis in A549 cells. The combined administration more effectively decreased the Bcl-2 expression and increased Bax gene expression in cells compared to ALA or PAX alone. Also, co-treatment of ALA and PAX caused apoptotic nuclear formations. Additionally, coadministration increased the caspase-3 and caspase-9 levels more than PAX or ALA alone. The increase in NF-κB gene expression levels suggests that an NF-κB-independent apoptotic trigger mechanism operates in cells. Together, the present in vitro findings suggest that ALA may contribute as a potential therapeutic strategy in the treatment of lung cancer.

**Supplementary Information:**

The online version contains supplementary material available at 10.1007/s00210-025-03947-w.

## Introduction

Non-small cell lung cancer (NSCLC) is the most common type, accounting for a large percentage (> 85%) of all lung cancer cases (Leiter et al. 2023). Chemotherapy, the most commonly used treatment for NSCLC, is unsatisfactory. Chemotherapeutic drugs may cause risky results in the long term due to drug resistance and irreversible side effects and may lead to failure to achieve the desired efficiency in treatment (Huang et al. 2017; Wang et al. 2019).

Paclitaxel (PAX)-like microtubule targeting agents (MTAs) lead to inhibition of cell mitosis and cell cycle arrest. MTAs suppress cell proliferation and can induce apoptosis in cancer cells (Liu et al. 2019; Sazonova et al. 2021; Zhao et al. 2022). PAX, a member of the taxane family, as a single agent, is an active drug in advanced NSCLC (Bernabeu et al. 2017). Results from phase II and III clinical trials have shown that PAX has some therapeutic efficacy on metastatic cancer types, including lung cancer (Chu et al. 2005; Yan-Hua et al. 2020). Taxanes, such as PAX, are an important part of chemotherapy regimens commonly used in metastatic/advanced NSCLC (Joshi et al. 2014). However, this antimitotic chemotherapeutics cannot act on apoptosis of cells that acquire drug resistance through different mechanisms, and the percentage of treatment success is significantly reduced (Liu et al. 2019; Das et al. 2021).

Alternative compounds with fewer side effects are needed to improve therapeutic efficacy in lung cancer. Natural products and their derivatives are important tools in this respect. Sesquiterpene lactones (SLs) are structures consisting of more than 5000 biologically active plant-derived compounds, especially belonging to the “Asteraceae” family (Gach et al. 2015; Babaei et al. 2018). SLs have been found to increase sensitivity to chemotherapeutic agents and reduce resistance to chemotherapeutic drugs through different mechanisms such as inhibition of NF-κB and STAT3 activation and ROS-mediated inhibition in different cancer cells (Fang et al. 2009; Maryam et al. 2017; Wang et al. 2019; Ding et al. 2022).

**Fig. 1:**
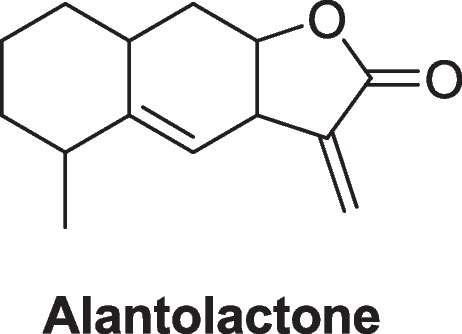
The chemical structure of alantolactone

Alantolactone (ALA), an important SL species, is an SL obtained from *Inula helenium* L. plant and has strong biological activities (anti-inflammatory, antihelminthic, anticancer, antifungal, etc.). The chemical structure of ALA is shown in Fig. 1. Studies have shown that ALA suppresses growth and development, induces apoptosis activation, and exhibits anticancer activity in different cancer cells (Khan et al. 2012; Maryam et al. 2017; Liu et al. 2018, 2019; Zhang et al. 2019).

Although the effects of ALA have been investigated in various human cancer cell lines to date, the mechanisms for the potential anticancer activity of its combined administration with PAX, an important clinical drug against human lung cancer, remain unclear. In the present study, we aimed to investigate the potential of ALA synergistically with PAX to suppress cell proliferation and induce apoptosis in A549 cells and the related molecular mechanisms. Our findings suggest that ALA has strong anticancer properties against lung cancer A549 cells and may be used as a booster agent for PAX treatment in lung cancer.

## Materials and methods

### Chemicals and materials

All chemicals used were commercially purchased from different companies.

### Cell culture

A549 human lung cancer cell line was purchased from the American Type Culture Collection (ATCC, USA) and cultured in Dulbecco’s Modified Eagle’s Medium (DMEM)-high glucose, supplemented with 10% fetal bovine serum (FBS), 1% GlutaMax, 100 U/ml penicillin, and 100 μg/ml streptomycin at 37 °C in an incubator with 5% CO_2_.

### Cell viability assay

Antiproliferative activity studies of ALA and PAX were performed according to the procedure described in the literature (Sahin et al. 2023; Mavvaji et al. 2023; Akkoc and Muhammed 2024; Bayar et al. 2024). The cell seeding was done at a density of 5 × 10^3^ cells/well into sterile 96-well plates. The cells were exposed to the molecules at 6.25, 12.5, 25, 50, 75, and 100 µM concentrations for 48 h. The MTT stock solution was added to the plate wells and was incubated for an additional 2 h. Absorbance values were measured in the Epoch 2 Elisa plate reader device at 590 nm. IC_50_ values were calculated using GraphPad Prism Software 5.

### Colony formation assay

Colony formation assay studies were performed as previously described (Erzurumlu et al. 2023a, b). Cells were seeded in a 6-well plate (1000 cells/ml). Twenty-four hours later, cells were treated with compounds and kept at 37 °C in a CO_2_ incubator for 72 h. Then, the culture media was removed, and the plate was rinsed with 1 × Dulbecco’s phosphate-buffered saline (D-PBS) twice. Colonies were fixed and stained with 0.05% crystal violet solution (Sigma-Aldrich). The inhibition of % colony growth was analyzed by the ImageJ program. Results were presented in the graph as % colonial growth.

### Wound healing assay

Cells were seeded in a 12-well plate at a density of 6 × 10^4^ cells per well. The plates were incubated for 24 h in a humidified incubator at 37 °C and 5% CO_2_. After a 24-h incubation period, wounds were formed on the cells, and the medium was removed. The cells were washed twice with PBS. Then, 1 mL of fresh medium was added to each well. PAX, ALA, or their combinations were not added to the first well. First well used as a negative control. Different concentrations of ALA, PAX, and their combinations were added to the other wells, respectively. Images of the cells were taken at 24 and 48 h using a Leica inverted microscope, and wound widths were measured with the Image-J program.

### Quantitative real-time PCR (qRT-PCR)

Gene expression analysis was performed as described before (Erzurumlu et al. 2023a, b). Total RNA was isolated from A549 cells using RiboEx™ (GeneAll) according to the manufacturer’s instructions. Complementary DNA was synthesized using 1 μg RNA by iScript™ cDNA Synthesis kit (Bio-Rad). The reaction mix was subjected to 5 min at 25 °C, 20 min at 46 °C, and 1 min at 95 °C, respectively. Primer sequences are available upon request. Quantitative real-time PCR amplification was performed by iTaq Universal SYBR® Green Supermix (Bio-Rad) according to the manufacturer’s instructions, and the fluorescence signal was detected by the CFX connect instrument (Bio-Rad). Relative mRNA expression levels of BAX (Forward: 5′-AAG AAG CTG AGC GAG TGT CT-3′, Reverse: 5′-TGG CAA AGT AGA AAA GGG CG-3′), BCL-2 (Forward: 5′-AGG ACA TTT GTT GGA GGG GT-3′, Reverse: 5′-AAA CGG AGC TGC ACT TTG AG-3′) and NF-κB (Forward: 5′-CGC ATC CAG ACC AAC AAC AA-3′, Reverse: 5′-GGG GCA CGA TTG TCA AAG AT-3′) were analyzed by qRT-PCR. Relative gene expression was normalized using housekeeping gene β-actin (Forward: 5′-AAA CTG GAA CGG TGA AGG TG-3′, Reverse: 5′-AGT GGG GTG GCT TTT AGG AT-3′) levels by the Livak method (Livak and Schmittgen 2001). Each cDNA sample was analyzed in triplicates for each qRT-PCR. The specificity of amplified PCR products was verified by melting curve analysis.

### DAPI staining

DAPI staining was performed as previously described (Erzurumlu and Catakli 2024). Cells were seeded in a 12-well plate (0.5 × 10^5^ cells/well) included in the cover glasses and were treated with compounds for 48 h. Growth media was aspirated and cells were fixed with a 4% formaldehyde solution. Thereafter, cells were blocked with bovine serum albumin (BSA) and stained with 4′,6-diamidino-2-phenylindole (DAPI) (Invitrogen™). Nuclear staining was visualized in a fluorescence microscope using a DAPI filter (Olympus EX71 microscope, DP74 camera system, Japan).

### Caspase-3 and caspase-9 enzyme-linked immunosorbent assay (ELISA)

The quantification of caspase-3 and caspase-9 levels was conducted by a commercial ELISA kit (Bioassay Technology Laboratory, Cat#4808Hu, Cat#E4994Hu) according to the manufacturer’s instructions. Basically, cells were homogenized with ice-cold 1xPBS (pH:7.4) at a ratio of 1:9 on ice, and the supernatant was collected. The supernatant (40 μl) or standards (50 μl) were added to 96-well plates, and then 10 μl antibodies and 50 μl streptavidin-HRP were added to the wells, respectively. The plate was incubated at 37 °C for 1 h. Following, the plate was washed with washing buffer five times, and then, substrate A and substrate B were added to the wells, respectively, and incubated at 37 °C for 10 min. Fifty microliters termination buffer was added, and optical density was measured at 450 nm in a spectrophotometer (BioTek, Epoch2). Caspase-3 and caspase-9 levels (ng/ml) were calculated according to the standard curve.

### Statistical analysis

Data were presented as means ± standard deviation. The statistical significance of differences between groups was determined by a two-tailed equal variance Student’s *t*-test with a minimum of 95% confidence interval or one-way ANOVA by GraphPad Prism 7. The significant level was set at 5% (*p* < 0.05) for all tests.

## Results

### Cytotoxicity of alantolactone (ALA) and paclitaxel (PAX) against A549 lung adenocarcinoma cells

**Fig. 2:**
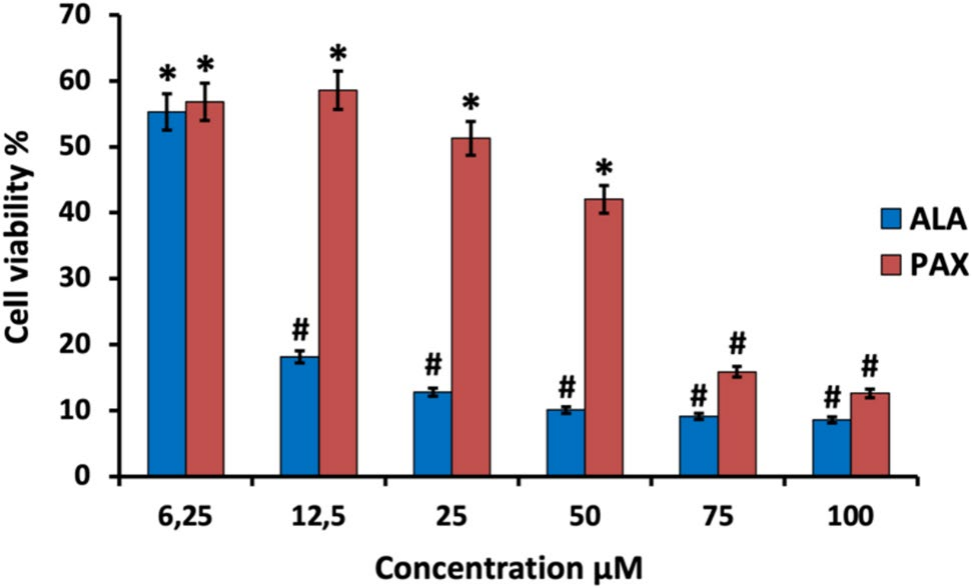
The cell viability rate (%) obtained as a result of 48-h incubation of A549 cancer cells in application groups with different concentrations of ALA and PAX (^*^p < 0.05, ^#^p < 0.001)

A549 cells were first treated with increasing doses (6.25–100 µM) of ALA and PAX for 48 h, and cell viability was evaluated by MTT analysis. ALA or PAX significantly reduced the viability of A549 cells in a dose-dependent manner (Fig. 2). IC_50_ values of ALA and PAX against A549 cells were determined as 5.32 µM and 32.30 µM, respectively. To characterize in detail the extent to which the combined treatment of ALA with PAX affects cell viability, coadministration was made to A549 cells at different determined concentrations, and cell viabilities were detected in the range of 14–27% (Supplementary data Fig. 1). In subsequent experimental studies, 5.3 μM and 2.16 μM ALA and 32 μM and 18 μM PAX and their combinations were used in A549 cells based on the result values.

### Effects of ALA and PAX on colony-forming abilities of A549 cells

**Fig. 3:**
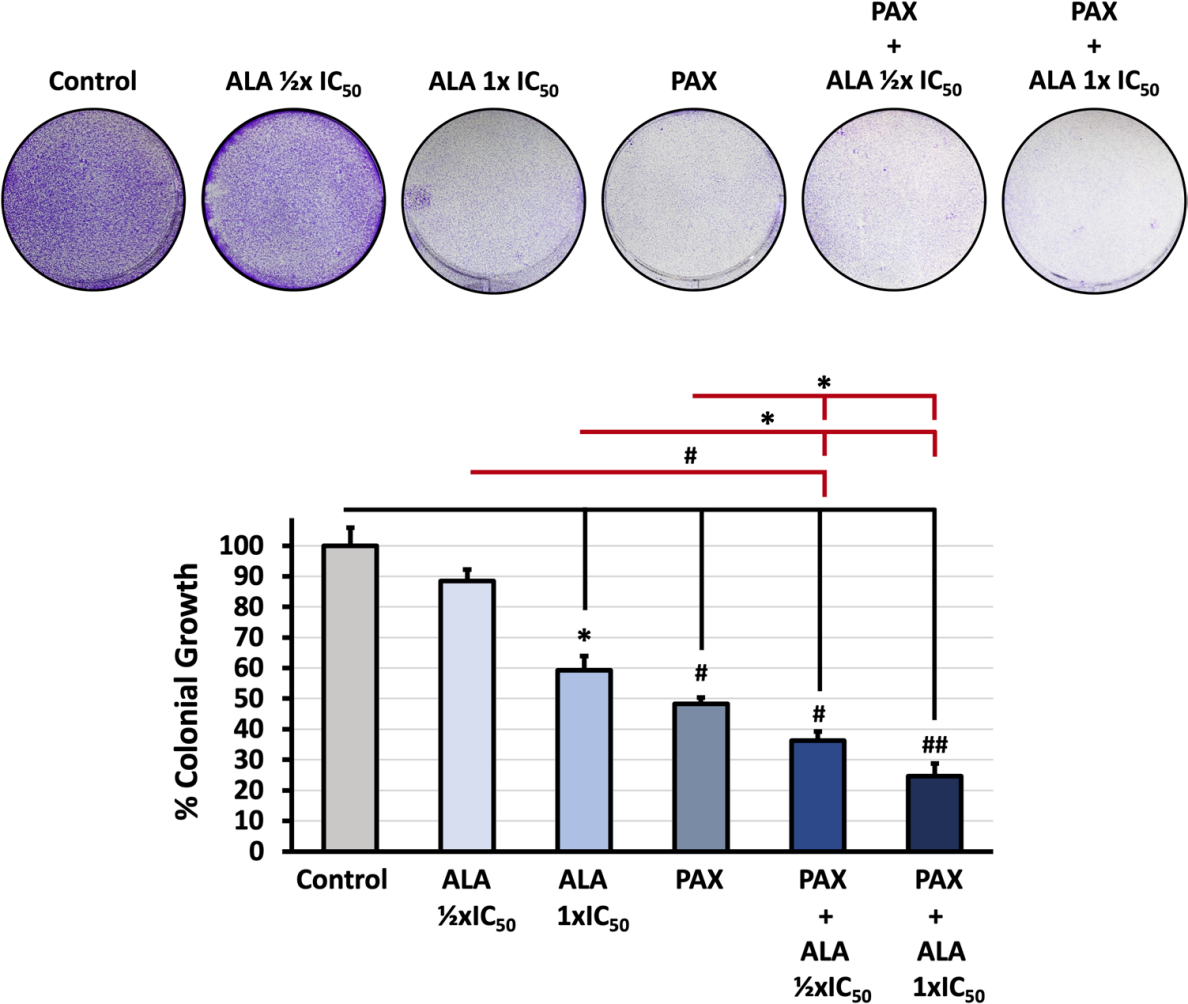
Evaluation of the effects of ALA and PAX applications on the colonic growth of A549 cells. Cells were treated with compounds for 72 h, and colonial growth was investigated by colony formation assay. The control was set to 100. Data presents the mean of three independent biological replicates in triplicates, and error bars represent SE as a fold change. Representative data was presented (^*^p < 0.05, ^#^*p* < 0.005, ^##^*p* < 0.001)

Colony formation assay was performed to examine the anti-proliferation effect of ALA and PAX in A549 cells (Fig. 3). The number of colonies in the treated groups decreased significantly compared to the control groups, respectively (*p < 0.05, #*p* < 0.005, ##*p* < 0.001). Compared to ALA or PAX applications alone, the lowest colony formation percentages were detected in the combined application groups (35% and 25%, respectively). The results revealed that the combined treatment exhibited strong inhibitory effects on cell proliferation of A549 cells.

### Effects of ALA and PAX on the migration ability of A549 cells

Wound healing analysis was performed for 24 and 48 h to examine the effect of ALA and PAX on migration in A549 cells. It was determined that migration decreased and wound widths increased in the ALA/PAX combined application groups compared to ALA alone (1 × IC_50_ and ½ × IC_50_) applications (*p* < 0.001) (Table 1). The results revealed that the combined treatment exhibited strong inhibitory effects on cell migration of A549 cells.

**Table 1:** Wound widths determined in cells treated with ALA and PAX

	Control	1 × IC_50_ ALA	½ × IC_50_ ALA	1 × IC_50_ PA ×	½ × IC_50_ PA ×	1 × IC_50_ ALA + 1 × IC_50_ PA ×	½ × IC_50_ ALA + ½ × IC_50_ PA ×
	Mean ± SE	Mean ± SE	Mean ± SE	Mean ± SE	Mean ± SE	Mean ± SE	Mean ± SE
24 h	923.67 ± 39.75^d^	713.00 ± 41.57^b^	411.67 ± 38.11^a^	732.00 ± 22.11^bc^	725.33 ± 15.41^bc^	730.33 ± 28.50^bc^	890.33 ± 52.88^ cd^
48 h	231.67 ± 15.03^a^	215.67 ± 15.38^a^	238.33 ± 46.67^a^	685.00 ± 6.11^c^	562.00 ± 33.86^bc^	508.00 ± 55.30^b^	513.33 ± 42.79^b^

The difference between the means with different letters on the same line is statistically significant at the *p* < 0.001 level

### Effect of ALA and PAX on Bcl-2, Bax, and NF-κB gene expression levels in A549 cells

**Fig. 4:**
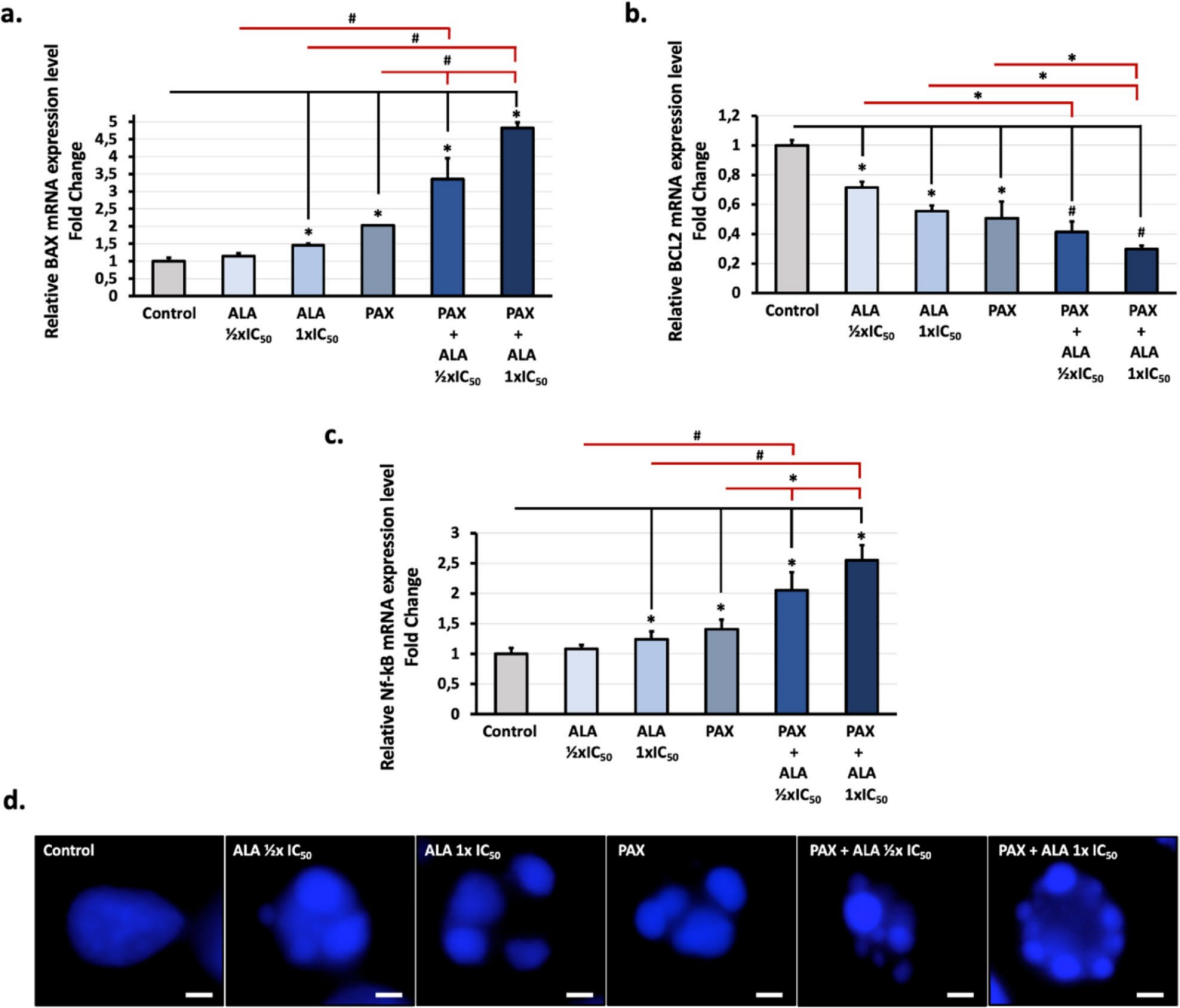
Investigation of the effects of ALA and PAX applications on mRNA levels of BAX, BCL2, and NF-κB and nuclear integrity in A549 cells. Cells were treated with compounds for 48 h and then relative mRNA expression levels of **a** BAX, **b** BCL2, and **c** NF-κB were analyzed by qRT-PCR. Beta-actin was used as a housekeeping gene. Control was set to 1. Data presents the mean of three independent biological replicates in triplicates, and error bars represent SE as a fold change. (^*^p < 0.05, ^#^*p* < 0.005). **d** Cells were treated with compounds for 48 h, and then, nuclear staining was performed using DAPI, as stated in the “Materials and method” section. Scale bar: 5 µm

The effects of ALA, PAX, and their combinations on the mRNA expression levels of anti-apoptotic Bcl-2 and pro-apoptotic Bax genes and the transcription factor NF-κB gene in A549 cells were analyzed by qRT-PCR. β-Actin was used as a reference gene. The results obtained showed that co-treatment applications led to effective BAX gene upregulation and Bcl-2 and NF-κB gene downregulation compared to ALA and PAX applications alone (Fig. 4).

### Effect of ALA and PAX on cellular morphological changes of A549 cells

In addition to experiments in which we tested BAX and BCL-2 expression to ascertain the effect of ALA and its treatment with PAX on apoptotic cell death in A549 cells, DAPI staining was performed, which showed remarkable changes in the nuclear morphology and membrane blebbing of these cells. Consistent with the qRT-PCR data, DAPI staining showed that ALA administration triggered the membrane blebbing and alteration of the nuclear structure similar to PAX alone compared to the control group. Treatment with PAX-ALA more strongly induced nuclear alterations in a dose-dependent manner and caused apoptotic bodies characterized by small disintegrated nuclear particles in A549 cells compared to ALA or PAX alone. Collectively, these results strongly suggest that ALA induced apoptotic cell death in A549 cells and combinator administration with PAX dose-dependently increased the anticancer effect of PAX on A549 cells (Fig. 4d).

### Effects of ALA and PAX on caspase-3 and caspase-9 levels in A549 cells

**Fig. 5:**
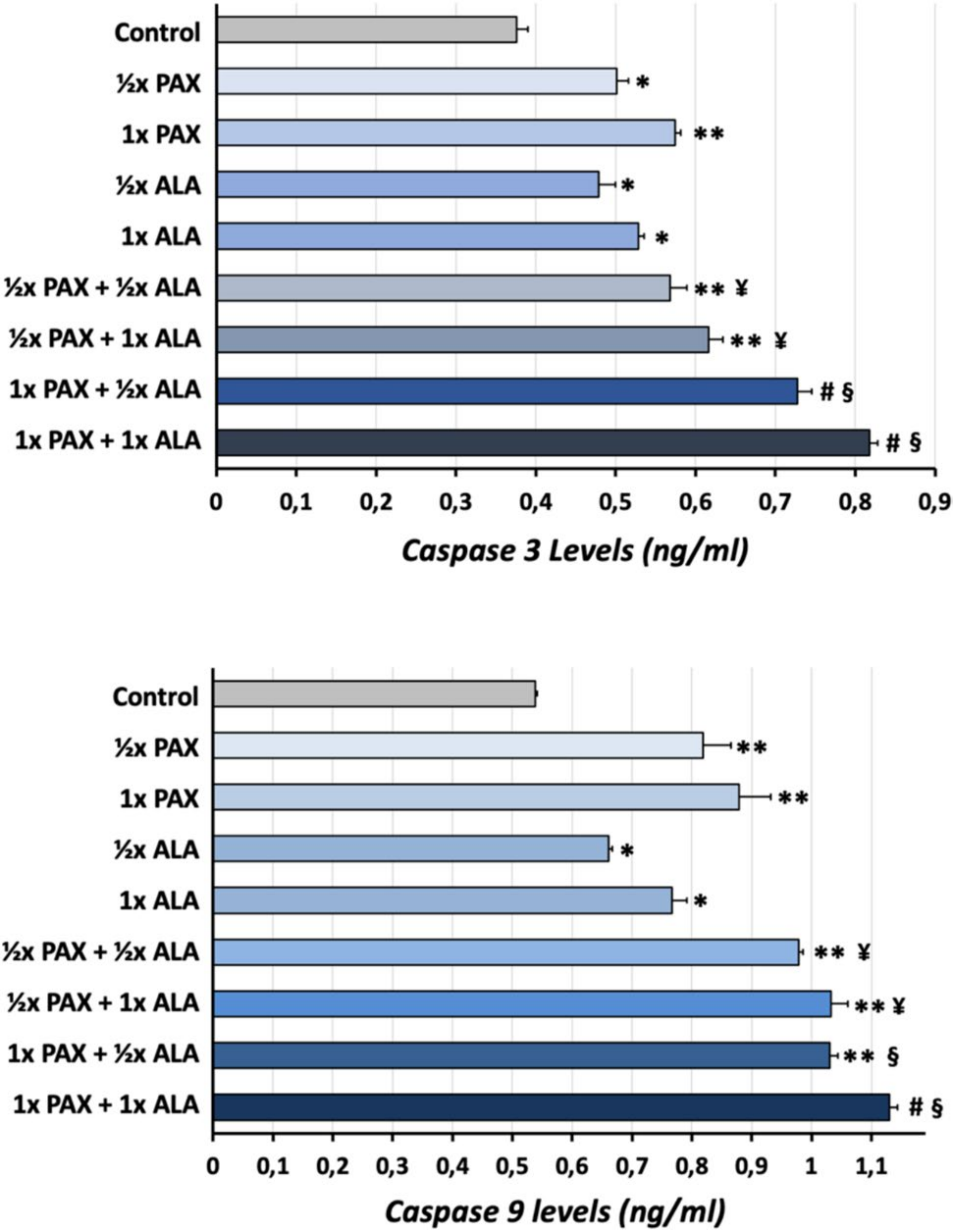
Evaluation of the effects of ALA and PAX applications on protein levels of caspase-3 and caspase-9 in A549 cells. Cells were treated with compounds for 48 h at 1 × IC_50_, ½ × IC_50_, and their combinations, and then caspase-3 and caspase-9 levels were determined by ELISA. (Groups compared to control, *p < 0.05, **p < 0.005, #*p* < 0.001; groups compared to ½ × PAX, ¥*p* < 0.05, §*p* < 0.005; groups compared to 1 × PAX, §*p* < 0.005)

The effects of ALA, PAX, and their co-treatments on apoptosis in A549 cells were evaluated by colorimetric analysis of caspase-3 and caspase-9 levels. The results obtained were parallel to the gene expression results, and it was determined that combination applications significantly increased (1.5–2.2 times) the apoptotic tendency compared to the control or PAX group (*p* < 0.05) (Fig. 5).

## Discussion

Lung cancer is one of the most commonly diagnosed cancer types in the world and is histologically divided into two main subtypes: small cell cancer (SCLC) and non-small cell cancer (NSCLC) (Li et al. 2023). PAX, a first-line chemotherapeutic drug, is a common treatment administered to millions of NSCLC patients worldwide (Bai et al. 2021). However, resistance to chemotherapeutic drugs and possible systemic side effects limit the success of treatment. Due to these limitations, research on using natural products to increase the effectiveness of existing treatments has been very popular for the last 20 years. Today, one of the crucial therapeutic strategies against cancer is to increase the sensitivity to conventional chemotherapy compounds with the help of natural compounds (Dehelean et al. 2021). ALA, an important SL compound obtained from *Inula helenium* L. (elecampane) root, has been reported to have anticancer activity (Babaei et al. 2021). The aim of this study was to determine the effects of ALA in combination with PAX on the suppression of cell proliferation and migration, induction of apoptosis, and NF-κB signaling pathway in A549 lung cancer cells.

ALA was observed to significantly inhibit the proliferation of A549 cells and induce apoptosis. It is clear that ALA has an antiproliferative effect in lung cancer cell lines (such as A549, NCI-H1299, and Anip-973) (Maryam et al. 2017; Liu et al. 2019; Ding et al. 2022). ALA was found to increase the antitumor effect of PAX on A549 cells. Combination treatments at different concentrations significantly reduced cell viability compared to PAX alone (Supplementary data 1, Supplementary Table 1).

Cell migration and colony formation in lung cancer are factors that can affect the prognosis of patients. We observed a dose-dependent decrease in colony formation and migration power of cells after treatment with ALA. We showed that treatment of ALA with PAX significantly inhibited the proliferative activity of cells and resulted in smaller colonies compared to single treatment (Fig. 3). Considering the systemic toxic effects of PAX despite its strong long-term efficacy, it is thought that the combined application of PAX with ALA may eliminate the toxic effects and strengthen the treatment efficacy by breaking the drug resistance of the cells.

Zhang et al. demonstrated that parthenolide, an important SL species, had a strong inhibitory effect on cell viability when treated with the chemotherapeutic agent Taxol on non-small cell lung cancer cell lines A549 and NCI-446 (Zhang et al. 2009). In another study, the combined application of parthenolide with oxaliplatin provided a strong antiproliferation effect in A549 cells (Fang et al. 2009).

Apoptosis, which is associated with the pathogenesis of cancer like many types of diseases, is a common target of anticancer treatments. In the apoptotic process that occurs through intrinsic (mitochondrial) and extrinsic pathways, the intrinsic pathway is regulated by the Bcl-2 protein family. It has been demonstrated that ALA induces apoptosis through the mitochondrial apoptotic pathway by significantly inhibiting Bcl-2 expression and increasing Bax expression in various cancer cell lines such as OSA, HepG2, and MCF-7 (Lei et al. 2012; Khan et al. 2012; Liu et al. 2018, 2019; Zhang et al. 2019). Our results showed that ALA upregulated Bax gene expression and downregulated Bcl-2 gene expression at increasing doses. However, ALA alone was not as successful as PAX in apoptosis induction. However, the synergistic effect of the two components showed that the apoptotic tendency increased significantly depending on the related gene expressions (Fig. 4). This is evidence that ALA potentiates PAX-induced cell apoptosis. DAPI analysis also confirmed the anticancer efficacy of the synergistic treatment (Fig. 4d).

Ding et al. (2022) investigated the anticancer activity of ALA in PAX-resistant A549 lung cancer cells. It was observed that ALA reduced cell viability and colonization in the relevant cells in a dose-dependent manner. The most effective concentrations of 5, 10, and 15 µM ALA were applied to A549 cells and were shown to induce apoptosis. The expression levels of PARP, caspase-9, and caspase-3 cleavage, which are apoptotic markers, were also confirmed to induce apoptosis. This is an important study that shows that SLs are promising agents for overcoming paclitaxel resistance in A549 lung cancer cells and also serves as a reference for our study (Ding et al. 2022).

Recent studies have shown that treatment of ALA with chemotherapeutic agents such as oxaliplatin and gemcitabine triggers apoptosis in colon cancer and lung cancer cells through different mechanisms (Cao et al. 2019; Wang et al. 2019). Activation of the apoptotic pathway results in caspase activation. In this context, in order to clearly evaluate the apoptotic tendency, ALA, PAX, and their co-treatments were applied to A549 cells, and the amounts of caspase-3 and caspase-9 were measured. The results are in line with Bcl-2 and Bax gene expression analyses, and there was a dose-dependent increase in caspase levels in cells treated with ALA and PAX. There was a significant increase in caspase-3 and caspase-9 levels in the combined application groups compared to the single applications (Fig. 5). These results suggested that ALA increased PAX-induced cell apoptosis in A549 cells. Although the caspase-3 and caspase-9 levels measured by the ELISA method do not target cleaved caspase versions but are for the determination of endogenous caspases, our findings suggest that the combined treatment of ALA and PAX induces the apoptotic process through caspase-3- and caspase-9-mediated stimulation. However, comprehensive analyses are needed to characterize further the apoptotic effect of ALA and PAX in A549 cells.

NF-κB has been identified as the most important survival factor in preventing apoptosis, and inhibition of this transcription factor can increase the effectiveness of cancer treatments that induce apoptosis (Beg and Baltimore 1996; Kucharczak et al. 2003; Dai et al. 2009; Sarkar and Li 2008). However, in our study, NF-κB gene expression results upregulated with the increase in ALA dose, contrary to the literature, and the highest NF-κB gene expression values were obtained in the combination groups. This may be an indication that apoptosis occurs independently of the NF-κB pathway. However, in order to reach a clear conclusion, it is thought that NF-κB protein and NF-κB inhibitor subunits IκBα, IκBβ protein and gene expressions should be evaluated together.

Previous studies reported that ALA elevated cellular reactive oxygen species (ROS) in several cancer cells (Nakatani et al. 2021; Nasirzadeh et al. 2023; Zhang and Zhang 2019). Also, it can suppress the cell cycle continuity in cancer cells (Zong et al. 2011). Similarly, many anticancer agents, including PAX, are known to induce apoptosis and lead to cell cycle arrest in cancer cells by increasing ROS levels (Nizami et al. 2023; Wang et al. 2000). In this context, present data thought that coadministration of ALA and PAX may exhibit more potent anticancer effects due to their joint impacts on ROS and cell cycle in A549 cells. Consistent with present data, in 2019, Wang et al. reported that ALA sensitized lung cancer cells to gemcitabine by increasing ROS production and inhibitory effects on the cell cycle (Wang et al. 2019). Additionally, considering that cancer cells have a shorter doubling time and increased levels of cellular oxidative stress, ALA may exhibit selective effects on lung cancer cells compared to normal cells (Maleki et al. 2024; Nakamura and Takada 2021). However, comparative and advanced mechanistic analyses need to confirm this hypothesis.

## Conclusion

In conclusion, the present study demonstrated that ALA enhanced the anticancer effect of PAX via apoptotic induction in A549 human lung cancer cells. These findings suggest that ALA exhibits potent synergistic activity with PAX and that such a combined therapy may be an effective clinical approach for the treatment of lung cancer in the future.

## Supplementary information

Below is the link to the electronic supplementary material.

Supplementary file1 (85.8 KB)

## Data Availability

All source data for this work (or generated in this study) are available upon reasonable request.
