# Supplementary material for: Investigation of the sensitivity of human A549 cells to paclitaxel and sesquiterpene lactone alantolactone via apoptosis induction
Source: Naunyn Schmiedebergs Arch Pharmacol. 2025 Feb 28;398(8):10625–33. doi: 10.1007/s00210-025-03947-w (PMC12350546; doi:10.1007/s00210-025-03947-w)
Supplement: Supplementary file 1 — Supplementary file1 (85.8 KB) [file 210_2025_3947_MOESM1_ESM.docx]

**Supplementary Data**


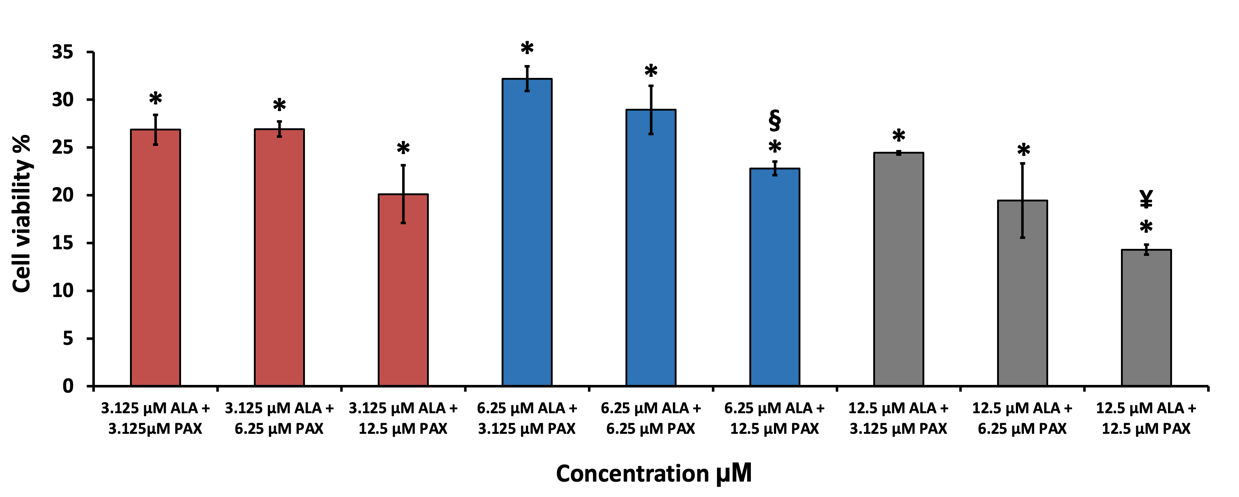


**Supple. Data 1.** % viability rates obtained as a result of 48-hour incubation of A549 cancer cells in coadministration groups with different concentrations of ALA (3.125, 6.25, 12.5 μM) and PAX (3.125, 6.25, 12.5 μM) (Groups compared to control ^*^p<0.001; 6.25 μM ALA + 3.125 μM PAX compared to 6.25 μM ALA + 12.5 μM PAX ^§^p<0.05; 12.5 μM ALA + 3.125 μM PAX compared to 12.5 μM ALA + 12.5 μM PAX ^¥^p<0.05).

**Supple. Table 1.** 48th hour % viability rates and statistical values of coadministration of ALA and PAX at different concentrations.

|  |  |
| --- | --- |
| Groups | **% Cell Viability** |
|  | **Mean ± SE** |
| 3.125 μM ALA + 3.125 μM PAX | 26.86 ± 1.56* |
| 3.125 μM ALA + 6.25 μM PAX | 26.93 ± 0.78* |
| 3.125 μM ALA + 12.5 μM PAX | 20.11 ± 1.96* |
| 6.25 μM ALA + 3.125 μM PAX | 32.19 ± 1.29* |
| 6.25 μM ALA + 6.25 μM PAX | 28.94 ± 2.51* |
| 6.25 μM ALA + 12.5 μM PAX | 22.81 ± 0.77*^,§^ |
| 12.5 μM ALA + 3.125 μM PAX | 24.43 ± 0.17* |
| 12.5 μM ALA + 6.25 μM PAX | 19.45 ± 3.88* |
| 12.5 μM ALA + 12.5 μM PAX | 14.28 ± 0.51*^,¥^ |

(Percent Viability = [ABS mean (each sample) / ABS mean (control)] x 100)

The difference between the means with different letters on the same line is statistically significant at the ^*^p<0.001 and ^§, ¥^ p<0.05.
